# Forensic profiling of non-volatile organic compounds in soil using ultra-performance liquid chromatography: a pilot study

**DOI:** 10.1080/20961790.2021.1899407

**Published:** 2021-07-12

**Authors:** Loong Chuen Lee, Ab Aziz Ishak, Ameeta A/P Nai Eyan, Anas Fahmi Zakaria, Nurul Syahiera Kharudin, Nor Azman Mohd Noor

**Affiliations:** a Program of Forensic Sciences, Centre for Diagnostic, Therapeutic & Investigative Studies, Faculty of Health Sciences, Universiti Kebangsaan Malaysia, Bangi, Malaysia; b Institute of IR 4.0, Universiti Kebangsaan Malaysia, Bangi, Malaysia; c Makmal Forensik Polis DiRaja Malaysia (PDRM), Cheras, Malaysia

**Keywords:** Forensic sciences, soil, non-volatile organic compound, liquid chromatography

## Abstract

Soil is of particular interest to the forensic community because it can be used as valuable associative evidence to link a suspect to a victim or a crime scene. Liquid chromatography is a powerful analytical tool for organic compound analysis. Recently, high-performance liquid chromatography (HPLC) has proven to be an efficient method for forensic soil analysis, especially in discriminating soils from proximity locations. However, ultra-performance liquid chromatography (UPLC), which is much more sensitive than HPLC, has never been explored in this context. This study proposed a UPLC method for profiling non-volatile organic compounds in three Malaysian soils (red, brown and yellowish-brown soils). The three soils were analysed separately to assess the effects of individual chromatographic parameters: (a) elution programme (isocratic *vs.* two gradient programmes); (b) flow rate (0.1 *vs.* 0.2 mL/min); (c) extraction solvent (acetonitrile *vs.* methanol) and (d) detection wavelength (230 *vs.* 254 nm). The injection volume and total run time were set to 5 µL and 35 min, respectively. Consequently, each soil sample gave 24 different chromatograms. Results showed that the most desirable chromatographic parameters were (a) isocratic elution; (b) flow rate at 0.2 mL/min and (c) acetonitrile extraction solvent. The proposed UPLC system is expected to be a feasible method for profiling non-volatile organic compounds in soil, and is more chemical-efficient than a comparable HPLC system.

## Key points

UPLC is a chemical-efficient analytical method for profiling non-volatile organic compounds in soil.

Mobile phase rich in acetonitrile promotes separation of non-volatile organic compounds in soil.

Isocratic elution is more desirable than gradient elution in producing a high-quality chromatogram.

## Introduction

Soil is a complex matrix and consists of loose inorganic and organic materials. By nature, soil is primarily composed of mineral particles [1]. However, a multitude of human activities have directly or indirectly contributed varying amounts of extraneous materials into soils. For example, organic materials are introduced into soils through the excessive use of fertilizers and pesticides by farmers [2, 3]. In contrast, heavy metal contamination in soils is typi­cally caused by industrial activities, such as mining and mineral processing [4, 5]. Therefore, soils origi­nating from different locations, regions or countries tend to be unique in composition. In practice, the evidential value of soil strongly depends on the diversity of the soil composition [6].

Numerous instrumental techniques have been applied for soil analysis and reported in numerous agricultural and geoscience studies [7–13]; however, optimizing the analytical methods for a very small quantity of sample is seldom a prime consideration. In a forensic context, the amount of soil that may be readily recoverable from a victim or suspect could be minute [14–17]. Hence, analytical methods that have the ability to acquire information from a minimal quantity of soil are the most-needed methods in forensic soil analysis.

Fundamentally, soil variability can be evaluated by physicochemical, chemical and biological properties [18–20]. Obviously, characterization of physicochemical properties including pH, moisture content, particle size distribution, colour, soil density and humic acid content [21, 22] is the simplest way to discriminate between soils. However, in practice, the comparison of soils by analysis of physicochemical properties alone seldom provides reliable discrimination [23]. To address this problem, various sophisticated instrumental techniques have been explored in the context of forensic soil science, mainly for studying the chemical composition of soil [24–29]. Recently, McCulloch et al. [30–32] published several articles regarding the feasibility of high-performance liquid chromatography (HPLC) in discriminating soils for forensic purposes. The authors concluded that HPLC has the potential to provide an accurate and practical method of differentiating soils originating from various proximity areas.

To date, there has been no report on the merits of advanced HPLC (i.e. ultra-performance liquid chromatography (UPLC)) in forensic soil characteri­zation. Technically, the particle size in a UPLC column (1.7 µm) is much smaller than that employed in HPLC systems (3–5 µm). Because of this diffe­rence, UPLC systems tend to outperform HPLC systems and show: (i) higher sensitivity, (ii) increased resolution, (iii) shorter analysis time and (iv) reduced consumption of eluent [33–35]. From this perspective, UPLC could be an alternative technique to HPLC for informative fingerprinting of soil and is especially advantageous when the amount of soil available for analysis is very small.

Therefore, the present study proposed a UPLC method to profile the non-volatile organic compounds of three Malaysian soils (red, brown and yellowish-brown soils). The soils were collected from the fern garden of the National University of Malaysia (Universiti Kebangsaan Malaysia; UKM). Four different UPLC parameters (elution programme, flow rate, detection wavelength and extraction solvent) were studied using the soil samples. The best UPLC chromatographic conditions were determined and then validated with respect to repeatability of peak retention time. The discrimination power of the proposed method is not reported herein but will be the topic of future work.

## Materials and methods

### Sample preparation

Three soil samples (red, brown and yellowish-brown soils) were collected from the fern garden of UKM, Malaysia. The soils were sampled following the grid-pattern procedure described by Pye [6], which was successfully applied by McCulloch et al. [30–32]. The procedure enables the researcher to assess the intra-location variability of the soil. First and foremost, locations showing one of the three soil colours were identified by a quick visual inspection. Then, a 1 m^2^ square grid was placed on the ground, and topsoil (0–10 cm) from the four corners and centrepoint of the grid was collected using a stainless-steel spatula. The other two soil samples were collected using the same procedures. The collected soil samples were transferred to the laboratory in separate clean labelled zip-lock plastic bags within a day of sampling.

The soil extraction method was based on the previous work of McCulloch et al. [30–32] with some modification. The samples were first dried in the laboratory at room temperature overnight. Before sieving through a stainless steel analytical sieve (600 µm), each sample was ground using a mortar and pestle. The fraction that passed through the sieve was placed in a glass petri dish and dried in an oven at 60 °C for 3 h. Dried soil (0.5 g) and 1.0 mL of HPLC-grade acetonitrile (ACN) (Fisher Chemical, Maharashtra, India) were placed into a 1.5-mL microcentrifuge tube. The snap cap of the tube was closed tightly before the tube was sonicated for 20 min. After the extraction step, the tubes were centrifuged at 13 000 rpm for 15 min. The supernatant (containing non-volatile organic compounds extracted from the soil) was carefully collected using a syringe and then filtered through an 0.2-µm polytetrafluoroethy­lene syringe filter into an HPLC vial.

To assess the impact of extraction solvent, another batch of extracts was prepared using the same extraction procedure using HPLC-grade methanol (Fisher Chemical) as solvent.

### UPLC analysis

UPLC was performed with a Waters ACQUITY UPLC^TM^ system (Waters Corp., Milford, MA, USA) equipped with a binary solvent manager, autosampler and photodiode-array detector (PDA). Chromatographic separation was carried out on a Waters ACQUITY UPLC^TM^ BEH C18 column (2.1 × 50 mm, 1.7 µm particle size). The mobile phase consisting of (A) water containing 10% ACN and (B) HPLC-grade ACN (Fisher Chemical) was used in three different elution programmes as shown in Table 1. The gradient-2 (G2) programme was used by McCulloch et al. [30–32], whereas the isocratic (ISO) programme was proposed by Bommarito et al. [24]. The gradient-1 (G1) programme is an adaptation from the G2 programme and is first reported in this work.

**Table 1.** Mobile phase compositions of the three elution programmes employed in this study.

Programme (ID)	Time (min)	% Water (A)	% Acetonitrile (B)
Isocratic (ISO)	–	35	65
Gradient-1 (G1)	Initial	60	40
	3.00	50	50
	5.00	20	80
	10.00	0	100
	25.00	0	100
	26.00	60	40
	30.00	60	40
Gradient-2 (G2)	Initial	53	47
	3.00	45	55
	24.00	26	74
	29.00	20	80
	31.00	20	80
	32.00	53	47
	35.00	53	47

Apart from comparisons of elution programme (ISO *vs.* G1 *vs.* G2 programme) and extraction solvent (ACN *vs.* methanol), this study also evaluated: (i) flow rate (0.1 *vs.* 0.2 mL/min) and (ii) detection wavelength (230 *vs.* 254 nm). In essence, this work has considered 24 different chromatographic conditions. The injection volume and total run time were fixed at 0.5 µL and 35 min, respectively, for each analysis. To avoid carryover, the autosampler needle and loop were purged with 600 µL of 10% ACN (weak wash), followed by 200 µL of 100% ACN (strong wash) before sample injection.

Data were collected using the built-in Empower^TM^ 2 software (Waters Corp.). The peak height and area were integrated by manually setting the baseline using the software.

### Statistical analysis

Principal component analysis (PCA) and hierarchical clustering analysis (HCA) are among the most popu­lar multivariate exploratory tools. Both techniques reduce the high dimensional chromatographic data and present variations of the data in graphical representations, such as score plots in PCA and dendrograms in HCA.

PCA constructs latent variables according to correlation/covariance among the exploratory variables [36–38]. In this work, the latent variables were constructed by considering all the peak area values reported in the chromatograms. Then, distribution among the samples (i.e. the 24 different chromatographic conditions for a particular soil sample) was inspected based on a two-dimensional score plot. Theoretically, similar chromatograms would be clustered together in the score plot and otherwise would be located far apart from each other.

In contrast, HCA employs multivariate distance metrics (e.g. Mahalanobis distance or Euclidean distance) to assess the similarities or dissimilarities among the samples [39]. The hierarchical relationship among the samples is typically illustrated in a dendrogram. Essentially, similar chromatograms are expected to be joined via a shorter distance in the dendrogram and otherwise would be connected *via* a greater distance.

In brief, PCA and HCA were employed to reveal information about (relative) variations of chromatograms for the four UPLC parameters of flow rate, extraction solvent, detection wavelength and elution programme. All statistical analysis was performed using the R programme for statistical computing (R version 3.6.2, https://cran.r-project.org/bin/windows/base/old/3.6.2).

## Results

The most desired UPLC chromatographic conditions were first determined by visual inspection on the 24 chromatograms generated for each of the three soil samples. Then, the interpretation derived from the visual examinations was further ascertained by analysis of the PCA score plot and the HCA dendrogram. For the sake of brevity, only the chromatograms of the red soil were discussed in detail whilst those of the other two soils were described briefly. Conversely, plots resulting from the two multivariate statistical tools were discussed for the three soil samples. The best UPLC chromatographic conditions were validated with respect to peak retention time repeatability.

### Visual examination of chromatograms

**Figure 1.**
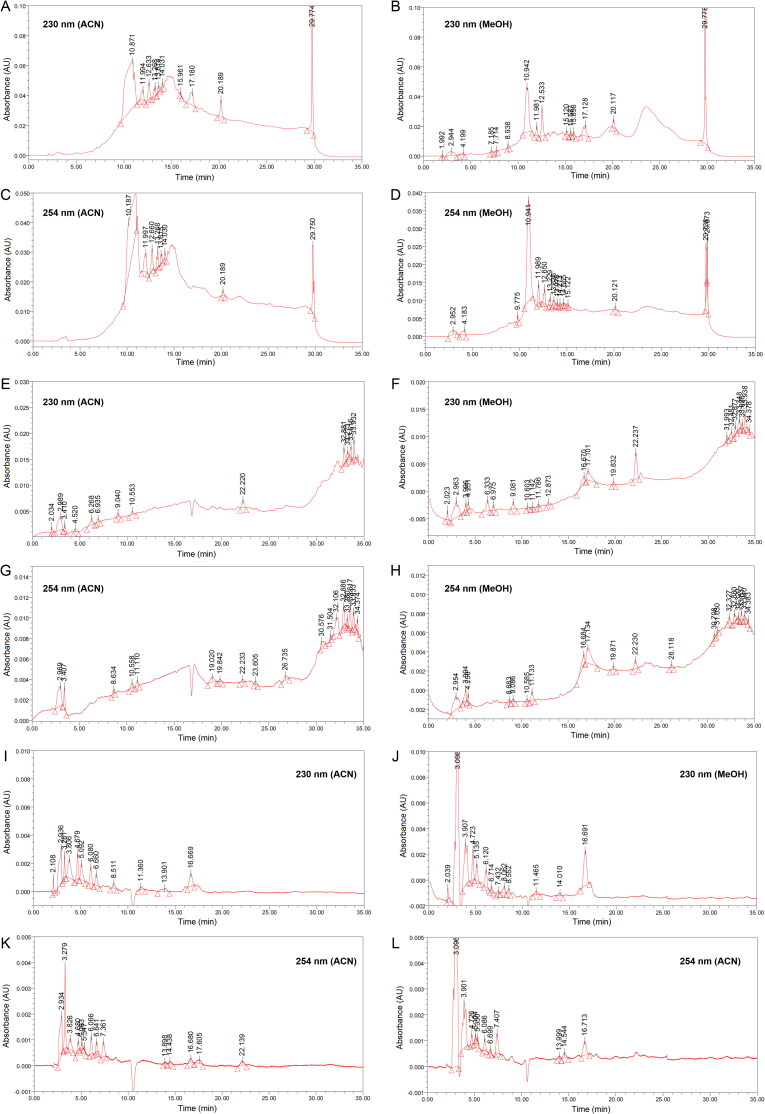
Chromatograms of red soil (flow rate 0.1 mL/min) using elution programme (A–D) gradient-1, (E–H) gradient-2 and (I–L) isocratic.

**Figure 2.**
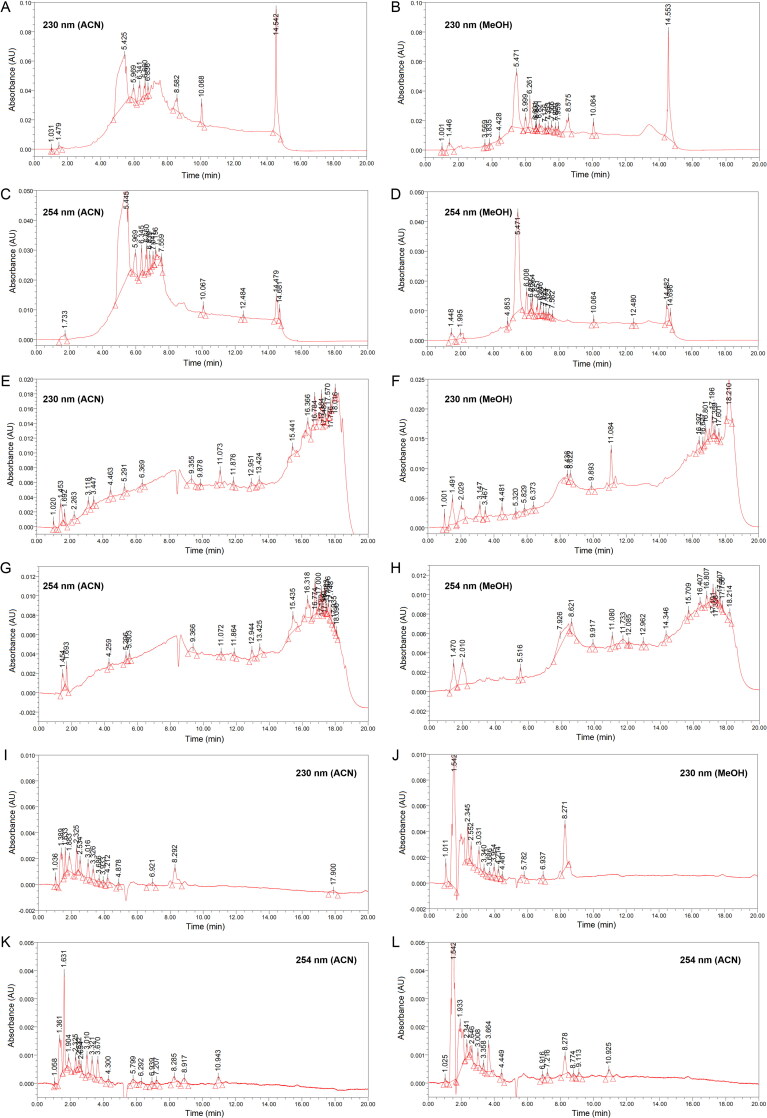
Chromatograms of red soil (flow rate 0.2 mL/min) using elution programme (A–D) gradient-1, (E–H) gradient-2 and (I–L) isocratic.

The chromatograms for red soil obtained at flow rates of 0.1 and 0.2 mL/min are presented in Figures 1 and 2, respectively. The chromatograms of the brown and yellowish-brown soil samples as well as the chromatograms of blank sample are available in Supplementary Figures S1–S5. Fundamentally, the blank sample did not contribute any significant peaks to the chromatograms of the soils. In addition, the quality of the peaks was not compromised by the rather small absolute response values presented by most of the chromatograms. Most of the peaks resembled typical Gaussian shapes [41].

In general, the chromatograms for red soil obtained using different elution programmes varied from each other, whereas flow rate, detection wavelength and extraction solvent caused only minor variations in the chromatograms. Thus, variation in absolute response value and in the overall chromatographic pattern were primarily influenced by the elution programme.

Although the absolute response values in the G1 chromatograms are rather high (0.10 AU), the respective chromatogram baselines were unstable (Figures 1A-D and 2A-D). In contrast, the lowest absolute response value was observed in the ISO chromatograms (0.005 AU). Nonetheless, baseline fluctuation was insignificant in the ISO chromatograms (Figures 1I-L and 2I-L). Theoretically, baseline drift is a common problem in gradient elution that is caused by poor column re-equilibration or differences in the absorbance properties of the mobile phases [41, 42]. Fundamentally, baseline refers to the part of the chromatogram having only mobile phase passing through the detector. Therefore, the baseline should be a straight line without visible peaks. This means that an unsteady baseline may render chromatogram data analysis and interpretation unreliable or inaccurate. Grounded on that principle, *isocratic elution is preferred over gradient elution*.

Chromatograms obtained at different wavelengths showed similar chromatographic patterns, although the absolute response values and the number of minor peaks showed variation. In general, chromatograms obtained at 254 nm showed slightly higher absolute response value than the corresponding chromatograms obtained at 230 nm. However, chromatograms obtained at 254 nm tended to present fewer peaks than chromatograms recorded at 230 nm. Overall, we considered that *both detection wavelengths are complementary to each other because each presented several peaks that are unique.*

Varying flow rates appeared to have little effect on overall chromatographic separation. By focusing on the ISO chromatograms, faster separation was achieved at 0.2 mL/min than at 0.1 mL/min. This was expected because 0.2 mL/min allows more mobile phase to pass through the column within the same period. Therefore, *the flow rate of 0.2 mL/min was selected in this study because it allows faster analysis while presenting a similar number of peaks and chromatographic pattern obtained using 0.1 mL/min.*

To examine the influence of the extraction solvent, only the ISO chromatograms were inspected carefully. This was because the ISO chromatograms were considered the most stable. Generally, the ACN extracts were preferred over the respective methanol extracts because the former produced steadier baselines. Therefore, *ACN was chosen because it resulted in less baseline noise.*

Mobile phase rich in ACN was observed to have more eluting power, and was able to separate more peaks. This can be observed in the G1 chromatograms in which most peaks were eluted in the region of 10–25 min (Figure 1A–D). Table 1 shows that this time interval used 100% ACN as the mobile phase. Thus, we considered that a *mobile phase rich in ACN promotes the chromatographic separation of soil sample components.*

The relative differences among the chromatograms of the brown and yellowish-brown soils were evaluated by referring to Supplementary Figures S1–S4. Overall, the variations caused by the parameters were similar to those observed in the chromatograms for red soil. Notably, the elution of non-volatile organic compounds in soil was improved by using mobile phase rich in ACN, especially when the ISO programme was adopted. However, the impact of flow rate was almost imperceptible except for the effect on total run time. Visual inspection suggested that *the best chromatographic conditions for the three soil samples were isocratic elution, detection at 230 and 254 nm, ACN extract, flow rate at 0.2 mL/min.*

It is noteworthy that chromatograms of the red, brown and yellowish-brown soils were different from each other. Red soil produced the most peaks, followed by brown soil and the yellowish-brown soil showed the fewest peaks. However, at this point, we emphasize that this work was aimed at establishing the best UPLC chromatographic parameters for qualitative profiling of non-volatile organic compounds in Malaysian soils rather than discriminating the three soil types.

### Multivariate exploratory examination

Typically, the purpose of multivariate exploratory tools like PCA and HCA is to discriminate or differentiate samples by origin or source [20, 43, 44]. However, in this work, PCA score plots and HCA dendrograms were employed to simultaneously illustrate the relative differences between the 24 chromatograms per soil sample in a two-dimensional space. This allowed us to verify the interpretation based on visual examination of the impacts of the four parameters (elution programme, extraction solvent, detection wavelength and flow rate) on the soil chromatograms. For this purpose, the 24 chromatograms of each soil sample were arranged in a matrix in which the rows and columns respectively represented the chromatograms and the peak area values.

**Figure 3.**
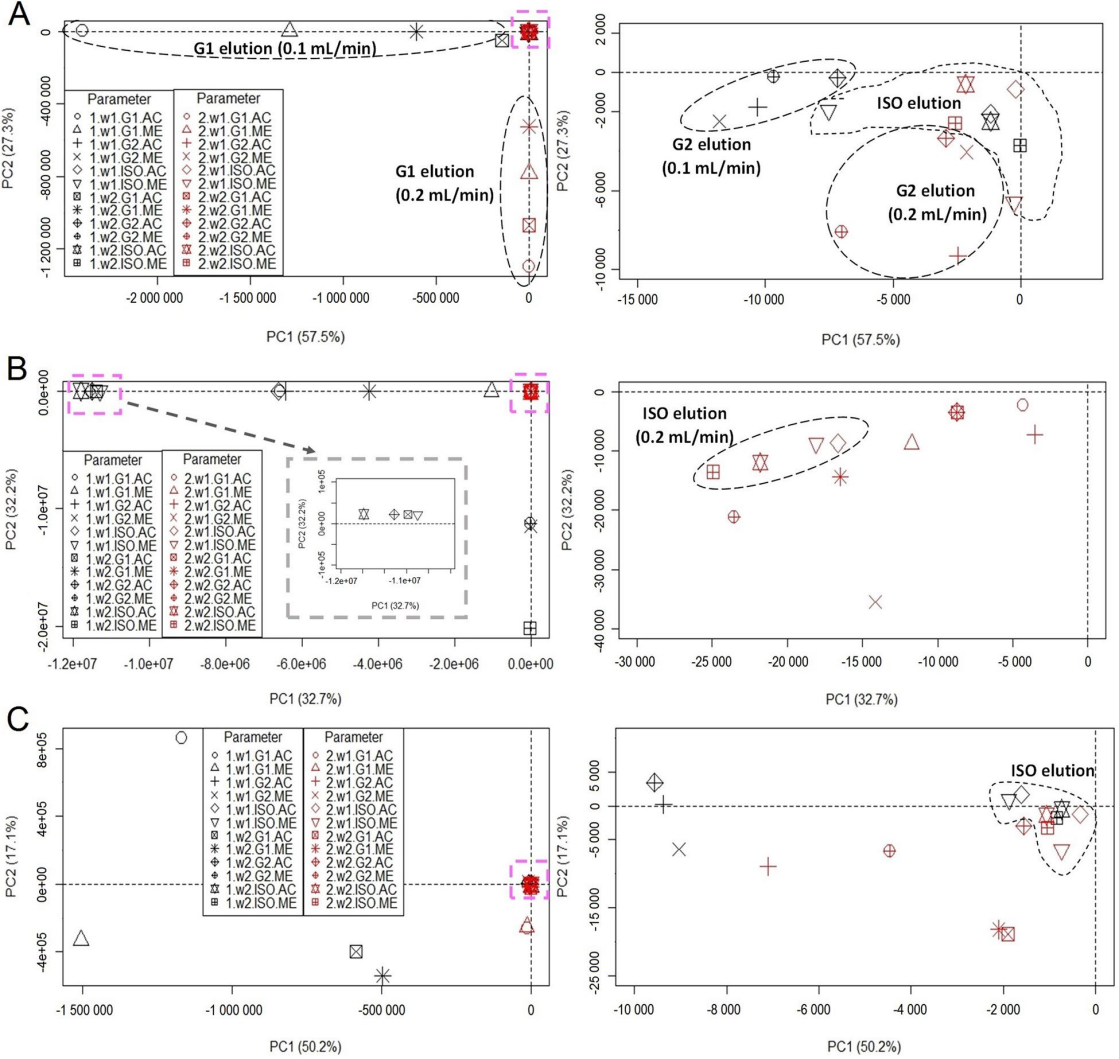
Score plots of 24 chromatograms of (A) red, (B) brown and (C) yellowish–brown soils, plotted on the first (PC1) and second (PC2) principal components. Each right plot is an expansion of the tight cluster (pink square box) in the left plot. Each point in the plots is labelled according to flow rate (1: 0.1 mL/min; 2: 0.2 mL/min), detection wavelength (w1: 230 nm; w2: 254 nm), elution programme (G1: gradient-1; G2: gradient-2; ISO: isocratic) and extraction solvent (AC: acetonitrile; ME: methanol).

Figure 3 shows the score plots generated using peak area values of the 24 chromatograms for the three soil samples. The tight clusters in the primary score plots (Figure 3, left panels, pink square boxes) are expanded and shown in the right panels of Figure 3. The corresponding dendrograms are shown in Supplementary Figure S6. The total explained variations represented by the first two principal components (PC1 and PC2) were about 85%, 65% and 67% for red, brown and yellowish-brown soils, respectively. Each score plot illustrated the similarities among the chromatograms because each plot accounted for more than 60% of the total variance.

In general, no soil samples formed visible clusters from the 24 chromatograms. However, careful inspection of Figure 3 revealed some of chromatograms were clustered according to elution programme, with a few chromatograms of red soil also clustered according to flow rate. However, it was difficult to identify clusters based on extraction solvent or detection wavelength. These findings are in line with visual examination, which suggested that elution programme was the primary factor causing differences among the 24 chromatograms of a given soil.

### Validation of best chromatographic conditions

The primary purpose of forensic soil analysis is to determine whether a particular soil shares a similar origin with a known soil. Typically, samples showing similar chromatographic profiles can be claimed to share similar origin. Therefore, quantitation and identification of compounds present in the soil are rarely the prime concern of the forensic soil analyst. In any case, the chromatographic method intended for comparative analysis must be validated with respect to retention time repeatability, reproducibi­lity and robustness [45–48]. Being a pilot study, we have not performed a well-planned validation experiment but have simply assessed the retention time repeatability, whereas the robustness of the UPLC method has been interpreted based on the variations observed in the PCA scores and HCA dendrogram.

The robustness of an analytical method refers to its capability to remain unaffected by small, intentional changes in the operational parameters of the method [45]. Typically, the development of a chromatographic method starts with the selection of column, eluent system and then the elution mode [42]. Because of technical constraints, this work considered only three different elution programmes using the same column. Therefore, the robustness of three elution programs was evaluated by varying the flow rate, detection wavelength and extraction solvent.

As shown in Figure 3, the ISO chromatograms were often clustered together and concentrated in the center (origin) of the score plots. This was especially true for yellowish-brown soil. However, G1 and G2 chromatograms, respectively, were scattered around in the score plots except for red soil samples. As a result, the ISO programme was considered more robust than the G1 and G2 programmes. The corresponding dendrogram (Supplementary Figure S6) suggests similar conclusions. In general, only chromatograms recorded *via* the ISO programme were connected *via* shorter distances. In contrast, chromatograms prepared using the gradient elution programmes, especially G1, were often joined *via* longer distances. All results suggested that the ISO programme was more robust than the two gradient programmes, and was unlikely to be affected by small changes in flow rate, detection wavelength and extraction solvent.

With the knowledge that the separation of non-volatile organic compounds in soil is promoted by a mobile phase rich in ACN, we performed another set of experiments utilizing an improved isocratic elution (i.e. ACN:H_2_O (9:1, v/v)), and the result was encouraging (data not shown). The derived optimum parameters for UPLC analysis of soil are shown in Table 2.

**Table 2.** Optimum UPLC parameters determined in this study for soil analysis.

Parameter	Details
Elution programme	Isocratic
Mobile phase	Acetonitrile:Water (9:1, v/v)
Column	ACQUITY UPLC BEH C18 column (2.1 × 50 mm, 1.7 µm particle size)
Extraction solvent	Acetonitrile
Flow rate	0.2 mL/min
Injection volume	7 µL
Total run time	15 min

The UPLC method was validated with respect to the repeatability of retention time. Relative standard deviation (RSD) is one of the most common metrics for evaluating retention time repeatability, and is mathematically defined by Equations (1–3).

(1) $$\text{RSD}\%=\frac{s}{\bar{x}}\times 100\%$$

(2) $$\bar{x}=\frac{1}{n}\sum_{i=1}^{n}x_{i}$$

(3) $$s=\sqrt{\frac{1}{\left(n-1\right)}\sum_{i=1}^{n}{\left(x_{i}-\bar{x}\right)}^{2}}$$

where *s* is standard deviation and x–$\bar{x}$ is the mean, as computed from n measurement values. A sequence of three consecutive injections was performed for each soil sample. As a result, each peak was represented by three measurement values. This allowed the assessment of the intra-day reliabi­lity (i.e. run-to-run repeatability) of the method based on the retention time of a peak in the soil sample.

To the best of our knowledge, previous studies have not reported the retention time repeatability [24, 27, 28, 30–32]. Therefore, an accepted reference value of RSD in forensic soil analysis is unavailable in the literature. This prompted us to adopt the benchmark value reported in typical pharmaceutical quality control work (1%) [48]. The results of precision experiments are presented in Tables 3 and 4, where the range of mean retention time of a peak was obtained based on the minimum and maximum values recorded for the red, brown and yellowish-brown soils. For all the components of the three soil samples, the RSD from three measurements was 0.02%–0.85%. With no RSD value above 1%, the UPLC method is regarded as highly reproducible.

**Table 3.** Run-to-run repeatability of retention time (detection wavelength: 230 nm).

Peak No.	Range of mean RT		RSD% (SD)		
	Min	Max	R	B	YB
1	0.954	0.968	0.22 (0.0021)	0.16 (0.0015)	0.10 (0.0010)
2	1.182	1.182	0.22 (0.0026)	–	–
3	1.640	1.643	–	0.07 (0.0012)	0.06 (0.0010)
4	1.700	1.718	0.09 (0.0015)	0.03 (0.0006)	0.17 (0.0030)
5	1.916	1.922	0.06 (0.0012)	0.11 (0.0021)	–
6	2.074	2.083	0.10 (0.0021)	0.03 (0.0006)	0.20 (0.0042)
7	2.226	2.226	–	0.12 (0.0026)	–
8	2.339	2.379	0.56 (0.0130)	0.15 (0.0035)	–
9	2.407	2.407	–	–	0.25 (0.0061)
10	2.492	2.538	0.25 (0.0062)	–	0.07 (0.0017)
11	2.732	2.735	–	0.35 (0.0095)	0.19 (0.0051)
12	2.878	2.893	0.22 (0.0062)	–	0.05 (0.0015)
13	2.937	2.937	–	0.29 (0.0085)	–
14	3.140	3.159	0.07 (0.0021)	–	–
15	3.396	3.472	–	0.46 (0.0159)	0.14 (0.0047)
16	3.556	3.584	0.14 (0.0050)	–	0.20 (0.0071)
17	3.998	4.093	–	0.39 (0.0158)	0.16 (0.0064)
18	4.565	4.609	0.12 (0.0053)	0.34 (0.0155)	0.17 (0.0076)
19	4.831	4.831	–	0.44 (0.0212)	–
20	5.043	5.083	0.22 (0.0113)	0.36 (0.0180)	0.19 (0.0095)
21	5.236	5.246	–	–	0.18 (0.0092)
22	5.356	5.400	0.15 (0.0079)	0.30 (0.0159)	0.16 (0.0087)
23	5.661	5.771	–	0.35 (0.0201)	0.13 (0.0075)
24	6.516	6.585	0.11 (0.0074)	0.35 (0.0228)	0.11 (0.0070)
25	6.776	6.852	0.14 (0.0096)	0.18 (0.0120)	0.15 (0.0106)
26	7.577	7.607	–	0.44 (0.0337)	0.03 (0.0026)
27	8.631	8.631	0.23 (0.0196)	–	–
28	8.680	8.713	–	0.14 (0.0123)	0.21 (0.0180)
29	9.093	9.093	–	0.47 (0.0424)	–
30	9.882	9.882	–	0.21 (0.0210)	–
31	10.646	10.646	–	0.11 (0.0121)	–
32	11.043	11.465	–	–	0.24 (0.0265)
33	12.149	12.216	–	0.17 (0.0202)	0.22 (0.0270)
34	12.856	12.856	–	–	0.85 (0.1089)
35	13.279	13.279	–	–	0.69 (0.0922)
Mean	–	–	0.18 (0.0065)	0.25 (0.0137)	0.21 (0.0157)
Min	–	–	0.06 (0.0012)	0.03 (0.0006)	0.03 (0.0010)
Max	–	–	0.56 (0.0196)	0.47 (0.0424)	0.85 (0.1089)

RT: retention time relative standard deviation; SD: standard deviation; R: red soil; B: brown soil; YB: yellowish–brown soil. The chromatograms were generated under the following conditions: extraction solvent: acetonitrile (ACN), flow rate: 0.2 mL/min, elution programme: ACN:H_2_O (9:1, v/v), injection volume: 7 µL, total run time: 15 min.

**Table 4.** Run–to–run repeatability of retention time (detection wavelength: 254 nm).

Peak No.	Range of mean RT		RSD% (SD)		
	Min	Max	R	B	YB
1	0.953	0.965	0.27 (0.0026)	0.16 (0.0015)	0.22 (0.0021)
2	1.639	1.642	0.14 (0.0023)	–	0.04 (0.0007)
3	1.733	1.742	0.12 (0.0021)	0.09 (0.0015)	0.09 (0.0015)
4	1.883	1.916	0.24 (0.0046)	0.08 (0.0015)	0.17 (0.0032)
5	2.009	2.009	–	–	0.08 (0.0015)
6	2.081	2.082	0.07 (0.0015)	0.15 (0.0031)	0.22 (0.0046)
7	2.216	2.216	–	0.11 (0.0025)	–
8	2.317	2.344	0.05 (0.0012)	0.07 (0.0015)	0.44 (0.0102)
9	2.486	2.486	0.08 (0.0021)	–	–
10	2.556	2.556	–	–	0.32 (0.0081)
11	2.732	2.747	–	0.29 (0.0080)	0.10 (0.0026)
12	2.875	2.882	0.25 (0.0071)	–	0.02 (0.0006)
13	2.945	3.081	0.39 (0.0120)	0.21 (0.0062)	0.12 (0.0036)
14	3.419	3.479	–	0.43 (0.0151)	0.14 (0.0049)
15	3.556	3.587	0.02 (0.0006)	–	0.31 (0.0112)
16	3.933	3.993	–	0.29 (0.0115)	0.25 (0.0101)
17	4.088	4.088	–	0.32 (0.0131)	–
18	4.199	4.220	0.43 (0.0180)	–	0.12 (0.0049)
19	4.545	4.590	–	0.36 (0.0165)	0.61 (0.0280)
20	4.937	4.937	–	0.47 (0.0232)	–
21	5.694	5.760	–	0.21 (0.0117)	0.16 (0.0090)
22	6.688	6.806	0.04 (0.0028)	0.42 (0.0281)	0.26 (0.0176)
23	7.543	7.619	–	–	0.13 (0.0095)
24	8.238	8.991	0.02 (0.0014)	–	–
25	9.122	9.790	0.09 (0.0085)	–	–
26	10.135	10.145	–	–	0.58 (0.0587)
27	11.879	11.879	0.02 (0.0028)	–	–
28	12.652	12.877	0.10 (0.0120)	0.18 (0.0230)	0.29 (0.0378)
29	13.215	13.278	–	–	0.11 (0.0146)
Mean	–	–	0.15 (0.0057)	0.24 (0.0118)	0.22 (0.0127)
Min	–	–	0.02 (0.0006)	0.07 (0.0015)	0.02 (0.0006)
Max	–	–	0.43 (0.0180)	0.47 (0.0281)	0.61 (0.0587)

RT: retention time; RSD: relative standard deviation; SD: standard deviation; R: red soil; B: brown soil; YB: yellowish–brown soil. The chromatograms were generated under the following conditions: extraction solvent: acetonitrile (ACN ), flow rate: 0.2 mL/min, elution programme: ACN : H_2_O (9:1, v/v), injection volume: 7 μL, total run time: 15 min.

**Figure 4.**
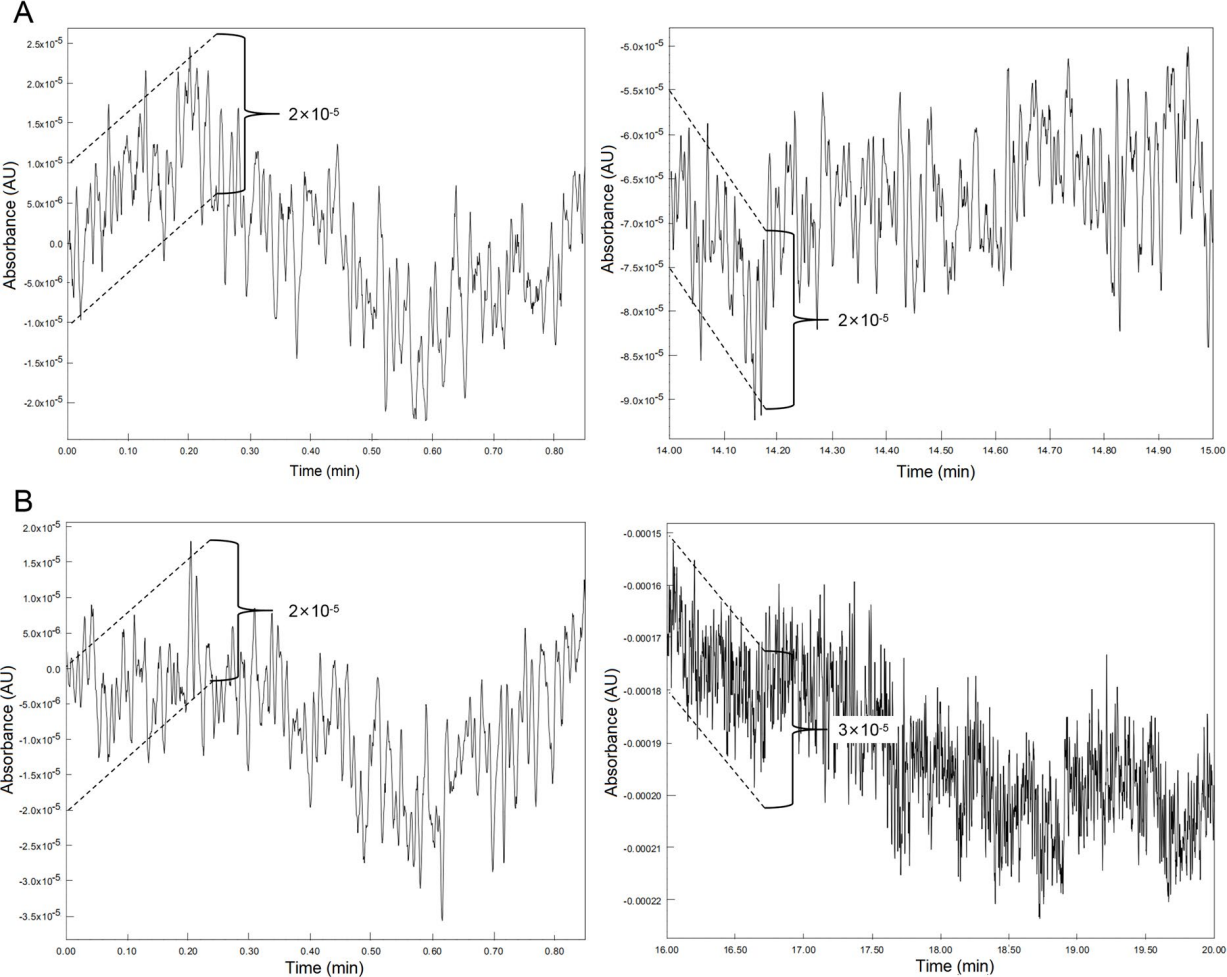
Amplified chromatogram baselines of a blank sample eluted using the ISO programme (0.2 mL/min) as detected at 230 nm (A) and 254 nm (B).

The effect of baseline noise is another important consideration in determining the reliability of chromatographic methods. To investigate the degree of baseline noise and disturbance, a blank run (no sample was injected) was performed and examined. It is important that the magnitude of baseline noise is at or below the accepted level. Figure 4 illustrates the amplified chromatographic baseline of the start and end regions for the blank sample. In general, the amplitude of the baseline ranged from 2 × 10^−5^ to 3 × 10^−5 ^AU, whereas the smallest signal of the chromatograms was value 1 × 10^−3 ^AU (Figures 2I-L and 3I-L). Hence, the peaks detected in Figures 2 and 3 are unlikely to be baseline noise.

## Discussion

To illustrate the merits of the UPLC system over the HPLC system, we should have observed the empirical performance of the HPLC system using the three Malaysian soils. However, technical constraints did not allow the collection of such data. McCulloch et al. [32] have also demonstrated that replication of previous work can be difficult because an identical column may not always be available. In facilitating the comparison, we have summarized the analytical parameters/performances of HPLC [24, 27, 28, 30–32] and UPLC (based on the current work) in Table 5.

**Table 5.** Comparison of HPLC and UPLC methods in forensic soil analysis.

Parameters	References				
	[27]	[28]	[24]	[30–32]	Current work
Method	HPLC	HPLC	HPLC	HPLC	UPLC
Sample drying (temperature/time)	Oven (65°C/2 h)	Oven (75 °C/1 h)	Oven (60 °C/2 h)	Air dry (NA/NA)	1. Air dry (NA/24 h) 2. Oven (60 °C/3 h)
Soil sample (g)	1.00	1.00	NA	0.25	0.5
Extraction solvent	10 mL ACN (HPLC grade)	10 mL MeOH	ACN	0.5 mL ACN	1.0 mL ACN (HPLC grade)
Sample conc. (g/mL)	0.1	0.5 (10 mL MeOH extract was evaporated into 0.5 mL)	1.0 (filtrate was evaporated to dryness, resolvated in ACN to make a 1 g/mL solution)	0.5	0.5
Sample processing steps	Vortex (10 min)	Vortex (10 min)	Sonicator (10 min)	1. Sonicator (20 min) 2. Centrifuge 13 000 rpm for 15 min	1. Sonicator (20 min) 2. Centrifuge 13 000 rpm for 15 min
Sample injection volume (µL)	5–20	NA	10	50	7
Column	Bondapk C_18_/Corasil (4.6 mm × 250 mm)	Bondapk C_18_/Corasil (4.6 mm × 200 mm)	C18 (5 µm,4.6 mm × 250 mm)	C18 (3.5 µm,4.6 mm × 150 mm)	C18 (1.7 µm,2.1 mm × 50 mm)
Particles size (µm)	NA	NA	5	3.5	1.7
Programme	Isocratic ACN:H_2_O (9:1)	Isocratic MeOH:H_2_O (8:2)	Isocratic ACN:H_2_O (65:35)	Gradient	Isocratic ACN:H_2_O (9:1)
Flow rate (mL/min)	2	NA	1	1	0.2
Run time (min)	15	NA	100	35	15
UV detection (nm)	254	230, 250, 270, 330	254	254	230, 254

NA: not available; ACN: acetonitrile; MeOH: methanol; HPLC: high-performance liquid chromatography; UPLC: ultra-performance liquid chromatography.

With respect to the minimum quantity of soil, this work extracted the non-volatile organic compounds in soil by mixing 0.5 g of soil with 1.0 mL of ACN. Although McCulloch et al. [30–32] successfully produced reliable chromatograms with only 0.25 g of soil, the final concentrations of the extracts were similar to those observed in the current work (∼0.5 g/mL). Therefore, we considered that the UPLC system was comparable with HPLC systems in terms of minimum soil sample size.

Although Reuland and Trinler [27] reported the use of injection volumes of 5–20 µL, the quality of the resulting chromatograms was not explicitly discussed. Therefore, we considered it is reasonable to assume the optimal injection volume for HPLC is 10–50 µL [24, 30–32]. Hence, it can be concluded that UPLC is more sensitive than HPLC because a reliable UPLC fingerprint could be produced from an injection of less than 10 µL.

Regarding run time, the current study found that 15 min could produce sufficient data for forensic soil analysis. In contrast, HPLC analysis required more than 15 min to obtain a good chromatogram from a soil sample [24, 28, 30–32]. Although Reuland and Trinler [27] reported that they needed only 15 min to complete chromatographic separation, details of retention time repeatability were not presented to the level demonstrated in the current study. As such, it is reasonable to conclude that the total run time to produce a reliable chromatogram is shorter for UPLC analysis than for HPLC analysis.

By minimizing injection volume and shortening total run time, the required volumes of extraction solvent and mobile phase are also reduced, which, in turn, decreases waste disposal costs. Although the purchase cost of a UPLC system (and column) is typically much higher than for an HPLC system, from a long-term perspective, UPLC consumes less eluent and will be much more chemical- and cost-efficient than HPLC.

The choice of extraction solvent is also an important consideration in optimising UPLC methods. In many cases, methods use an extraction solvent that is also a component of the mobile phase (Table 5). Theoretically, equilibrium between the sample and the mobile phase can be instantaneous if the extraction solvent is part of the mobile phase. Furthermore, given the preference for ACN over methanol in the literature [49, 50], only ACN/water mixture was used in this pilot study.

Although it is generally agreed that spatial cha­racterization is an important aspect in any soil-based analysis [51], this work has not considered temporal and spatial variations as reported by Bommarito et al. [24] and McCulloch et al. [32]. These aspects will be considered when we assess the discrimination power of the method in a further empirical study.

## Conclusion

This study describes the first application of a UPLC–PDA technique for the profiling of non-volatile organic compounds in Malaysian soils. When compared with HPLC, UPLC reduces analysis time, injection volume and solvent consumption. These saving characteristics of UPLC can potentially double the productivity of the method. Isocratic elution employing an ACN-rich mobile system is recommended for obtaining a high-quality chromatogram that provides good retention time repeatability. In addition, strong preference is given to an extraction solvent that also serves as a component of the mobile phase. In further work, larger and more diverse soil samples should be used to confirm the merits of the UPLC method, and spatial and temporal variations must be considered.
